# Cluster Analysis as a Statistical Method for Planning the Optimal Placement of Automated External Defibrillators

**DOI:** 10.3390/jcm14165686

**Published:** 2025-08-11

**Authors:** Rafał Milewski, Jolanta Lewko, Magda Orzechowska, Agnieszka Lankau, Anna Baranowska, Beata Kowalewska, Robert Milewski, Mateusz Cybulski

**Affiliations:** 1 Department of Integrated Medical Care, Medical University of Bialystok, 15-096 Bialystok, Poland; magda.orzechowska@umb.edu.pl (M.O.); agnieszka.lankau@umb.edu.pl (A.L.); anna.baranowska@umb.edu.pl (A.B.); beata.kowalewska@umb.edu.pl (B.K.); mateusz.cybulski@umb.edu.pl (M.C.); 2 Department of Primary Health Care, Medical University of Bialystok, 15-096 Bialystok, Poland; 3 Department of Biostatistics and Medical Informatics, Medical University of Bialystok, 15-096 Bialystok, Poland; robert.milewski@umb.edu.pl

**Keywords:** AED, OHCA, public health, cluster analysis

## Abstract

**Background/Objectives:** Out-of-hospital cardiac arrest (OHCA) remains a major public health challenge, with survival rates significantly dependent on early defibrillation. In Bialystok, Poland, the bystander usage rate of automated external defibrillators (AEDs) is extremely low, and the current distribution of public-access AEDs may not support optimal response times. The aim of this study was to identify an effective AED placement strategy using spatial analysis. **Methods:** We retrospectively analyzed 49,649 emergency dispatch records from 2018 to 2019, identifying 787 patients with OHCA within Bialystok’s city limits. After excluding ineligible records, 766 cases were geolocated and subjected to cluster analysis using the K-means algorithm. The goal was to determine optimal AED locations based on the geographic distribution of OHCA cases in both public and residential settings. **Results:** AEDs were used in only 0.51% of all cases of OHCA. Most cardiac arrests occurred in private homes (80.05% of cases). Cluster analysis identified 18 to 36 optimal AED locations, revealing significant mismatches with the current AED network. Notably, grocery store chain “PSS Spolem” emerged as an ideal AED deployment partner due to alignment with identified high-incidence clusters. **Conclusions:** The current AED distribution in Bialystok is inadequate for an effective response to OHCA. Geographic cluster analysis can significantly improve placement strategies. Priority should be given to residential areas and commonly accessed sites. Enhanced public education, a national AED registry, and improved accessibility are essential for increasing AED use and survival rates.

## 1. Introduction

According to available data, the incidence of out-of-hospital cardiac arrest (OHCA) in Poland is approximately 170 cases per 100,000 individuals annually [1]. OHCA results in the sudden cessation of mechanical cardiac activity, leading to a halt in blood circulation, secondary respiratory failure, and, ultimately, irreversible cerebral damage. If left untreated, OHCA inevitably leads to death. Four primary cardiac arrest rhythms are recognized: asystole and pulseless electrical activity (PEA), which are non-shockable, and ventricular fibrillation (VF) and pulseless ventricular tachycardia (pVT), which are shockable and require defibrillation to restore normal cardiac function [2]. VF is most commonly observed during the initial minutes of cardiac arrest [3] and is considered the rhythm associated with the most favorable prognosis in this patient group [4]. Early initiation of cardiopulmonary resuscitation (CPR) and prompt defibrillation significantly increase the chances of survival [5]. According to the European Resuscitation Council (ERC), when defibrillation is performed within the first 3–5 min of cardiac arrest, survival rates can reach 50–70% [3]. However, each minute of delay in defibrillation decreases the probability of survival to hospital discharge by approximately 10% [3,6].

Given that emergency medical services (EMS) response times are often too long, typically ranging from 5 to 8 min [7,8,9] and the time to first defibrillation may extend to 8–11 min [10,11], immediate intervention by a bystander is crucial for patient survival. Currently, EMS systems worldwide are not equipped to ensure that every patient with OHCA receives defibrillation within the critical first few minutes of cardiac arrest. Nevertheless, early defibrillation is possible using publicly accessible automated external defibrillators (AEDs) that can be used by bystanders. To increase accessibility and enable rapid defibrillation, initiatives to expand the availability of AEDs have been undertaken [12].

As of 1 February 2023, there were 2499 reported AED locations in Poland, according to data from www.ratujzsercem.pl (accessed on 1 February 2023). This figure does not reflect the total number of AED devices, as some facilities may possess more than one unit, and there is no formal registry providing an exact count of AEDs nationwide [13].

For many years, countries around the world have been implementing Public Access Defibrillation (PAD) programs aimed at ensuring prompt access to AEDs by bystanders. The installation of AEDs in public spaces not only enhances the sense of safety but also facilitates the provision of immediate assistance to individuals in need, benefiting both residents and tourists.

According to the 2021 ERC guidelines, AEDs should be installed in locations where at least one OHCA has occurred within the past five years. They should be positioned in high-traffic areas where the likelihood of prompt recognition of cardiac arrest is high. In residential areas, past incidents and specific characteristics of the neighborhood should inform AED placement decisions. Publicly accessible AEDs should be available to bystanders 24 h a day [3,14,15]. Furthermore, the ERC emphasizes that analysis of historical cardiac arrest data in a given region, along with an assessment of the area’s demographic and functional characteristics, is essential for effective AED deployment planning [3].

The aim of this study was to analyze OHCA cases in the city of Bialystok and to develop a map indicating the optimal distribution of AEDs. This approach may contribute to improving survival rates and enhancing the safety of residents and other individuals present in the city.

## 2. Materials and Methods

### 2.1. Patients

**Figure 1:**
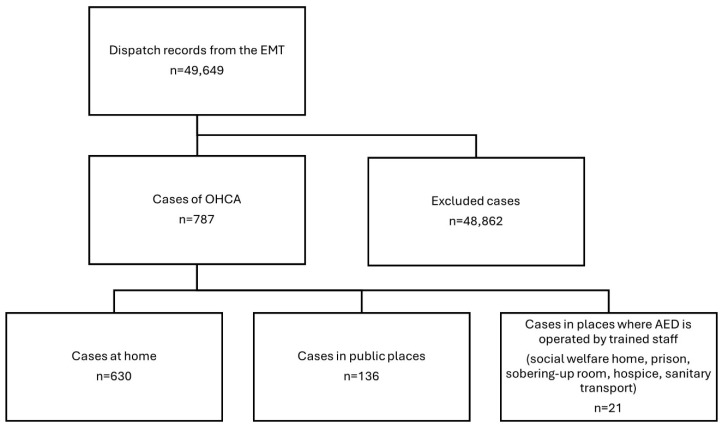
Flow chart showing the number of OHCA cases.

The dataset analyzed in this study comprised 49,649 dispatch records collected over a two-year period (2018–2019) by the EMS of the Voivodeship Emergency Medical Station in Bialystok. The research was confined to the administrative boundaries of the City of Bialystok, which spans an area of 102.13 km^2^ and has an estimated population of approximately 298,000 residents [16]. Inclusion criteria encompassed cases in which cardiac arrest occurred either prior to the arrival of EMS personnel or during the provision of prehospital medical care, as identified by specific ICD-10 codes (I46, I49.0, R96, R98, R99). A total of 787 patients fulfilled the criteria and were included in the analysis. Records were excluded if they contained ICD-10 codes outside the defined scope or if the dispatch documentation was incomplete or recorded incorrectly (Figure 1).

Data were collected using a self-administered questionnaire completed by the researcher. The collected data were analyzed, and the required assumptions were verified.

### 2.2. Statistical Analysis

Geographic coordinates of the OHCA cases were extracted from the dispatch records and verified using GIS software “GeoDa”. Coordinates were input as latitude-longitude pairs into the K-means clustering algorithm. The method optimizes the intra-cluster distance without considering road network constraints, which is a limitation. No socio-demographic or infrastructure layers were included in the model. To analyze spatial patterns, we employed K-means clustering, a non-inferential algorithm for identifying geographical groupings. We used K = 18 to reflect the actual number of publicly accessible AEDs in Bialystok and K = 36 to simulate a scenario in which the AED network was doubled. These values were selected to represent the current state and a realistic strategy for enhancing public AED coverage. Although no systematic sensitivity analysis of K was performed, these two settings provided useful comparative insights for public health planning. For inferential purposes, a chi-square test was used to examine associations between cardiac rhythm types and clinical outcomes such as return of spontaneous circulation (ROSC) and hospital transport. Statistical significance was set at *p* < 0.05. Data were compiled using Microsoft Office Excel and subsequently subjected to statistical analysis using STATISTICA 13.3 software (TIBCO Software Inc., Palo Alto, CA, USA).

### 2.3. Bioethics Committee Approval

The Bioethics Committee of the Medical University of Bialystok approved this study (No. R-I-002/610/2018, 20 December 2018).

## 3. Results

### 3.1. Previous Use of AEDs in Bialystok

Our previous study demonstrated that the use of AEDs prior to the arrival of EMS in Bialystok was rare. Over a two-year period, AEDs were used only four times out of 787 total OHCA cases (0.51%). In the majority of incidents (62.39%, 491 cases), no AED was used. In 37.10% of cases (292 records), the medical documentation lacked information to determine whether defibrillation had been performed. Of the four documented AED uses, two were administered by firefighters, one by detention facility staff, and one by sobering station personnel. Importantly, no AEDs were used by lay bystanders, that is, individuals without formal access to or possession of the device, during 2018–2019. Furthermore, a statistically significant relationship was identified between the presence of a shockable rhythm during the initial rhythm assessment by EMS and both the occurrence of ROSC and hospital transfer. Among patients with an initial rhythm of VF/pVT, ROSC was achieved in 58.62% of cases. In contrast, among patients presenting with non-shockable rhythms (asystole and PEA), ROSC occurred in only 26.56% of cases. This association was confirmed using a chi-square test (*p* < 0.0001), indicating a statistically significant relationship. These findings prompted the present study, which aimed to improve survival outcomes for patients with OHCA in Bialystok.

### 3.2. Incidence and Location of OHCA Cases

In Bialystok, the incidence of OHCA is 133.1 per 100,000 inhabitants per year. When considering only those cases in which EMS performed CPR, the rate is 67.3 per 100,000 residents annually.

The vast majority of OHCA incidents occurred in private homes or apartments (630 out of 787 cases, 80.05%). Only about one in ten patients (9.78%, 77 cases) experienced OHCA on the street. The remaining 10.17% (80 cases) occurred in the following locations: nursing homes (12), shops (8), outpatient clinics and construction sites (6 each), hotels (5), sobering stations, detention centers, allotment gardens, parks, beaches (3 each), garages, workplaces, sewage treatment plants, car repair shops, homeless shelters, and transport ambulances (2 each). Single cases occurred in shopping malls, train and bus stations, universities, dormitories, schools, government offices, libraries, sports stadiums, wedding venues, wholesale markets, hospices, abandoned buildings, forest areas, and trash shelters.

### 3.3. Profile of EMS Callers

Calls to the EMS for victims of OHCA in Bialystok were made by a variety of individuals. The most frequent callers were the family members of the patient (67.47%, 531 cases). EMS was less frequently summoned by bystanders (11.94%, 94 cases) or by uniformed services such as the police, fire department, city guards, or other EMS units (4.96%, 39 cases). The remaining 15.63% (123 cases) involved callers such as neighbors, partners, friends, roommates, caregivers, staff from nursing homes, homeless shelters, retail outlets, teachers, outpatient clinic staff, night and holiday health care providers, personnel from detention centers, sobering stations, hospices, hotel receptionists, or the patient themselves.

### 3.4. Reason for EMS Activation

In cases of OHCA, EMS was not always dispatched due to an explicitly stated cardiac arrest. Dispatcher interviews with callers enabled identification of the main reasons for EMS activation. The most common reason was the absence of breathing, strongly suggesting cardiac arrest (41.80%, 329 cases). Unconsciousness with preserved breathing accounted for 17.92% (141 patients). Other reasons included dyspnea (10.80%—85 cases), syncope (6.86%—54), and chest pain (3.05%—24). A variety of additional causes (19.57%—154 cases) included agonal respiration and lack of contact (24 each), oral bleeding/hematemesis (14), seizures and choking (10 each), hanging (9), unresponsive patients and window/balcony falls (7 each), diabetes and slurred speech (5 each), drowning (4), psychiatric disorders, traffic accidents, pedestrian injuries, altered consciousness (3 each), fire, hypotension, head trauma, and falls from height (2 each), as well as beating, gynecological hemorrhage, arrhythmia, bleeding victim, cyanosis, drug overdose, train strikes, general malaise, homicide by suffocation, being buried under soil, stabbing, self-inflicted lacerations, hypothermia, and falls from stairs (1 case each).

Overall, OHCA accounted for 1.6% of all EMS dispatches in Bialystok.

### 3.5. AED Network in Bialystok

Based on data from the Bialystok City Hall, AED maintenance companies (AEDMAX, Ratuj Z Sercem), and our own field research, an updated list of AED devices in Bialystok was compiled and personally verified as of 1 February 2023.

Currently, Bialystok has 18 so-called “public” AED units, accessible to all individuals, including untrained bystanders, and are installed outside buildings. These devices are available 24/7, except for those located on elementary school premises, where access is limited to school opening hours.

Additionally, 28 AEDs are available in locations not accessible to the general public. These devices are installed within private businesses, retail stores, workplaces, and government institutions and are primarily designated for use by on-site personnel trained or assigned to operate them.

#### AED Placement Optimization and Cluster Analysis

To determine the optimal AED placement strategy, 21 OHCA cases in which AEDs were used by trained personnel were excluded from further analysis, leaving 766 OHCA cases for spatial evaluation. The precise geographic coordinates (latitude and longitude) of each incident were extracted from EMS dispatch records.

A cluster analysis was conducted using the K-means method applied to two datasets: (1) all OHCA incidents, both in public and residential locations (*n* = 766), and (2) OHCA incidents occurring exclusively in public spaces (*n* = 136). This method facilitated optimal allocation of AEDs by minimizing the distance between OHCA occurrence sites and the nearest AED.

For each group, a spatial map was generated, and optimal AED placement points were identified. In the first scenario, 18 locations were proposed for either relocation of existing public AEDs or addition of 18 new devices alongside current ones. In the second scenario, assuming a doubling of the existing network, 36 optimal points were designated, either by relocating 18 existing units and adding 18 more or by keeping all current units in place and suggesting 36 additional optimal locations. The comparative analysis was based exclusively on publicly accessible AED devices.

OHCA cases in Public and Residential Locations (Number of Clusters = 18).

**Figure 2:**
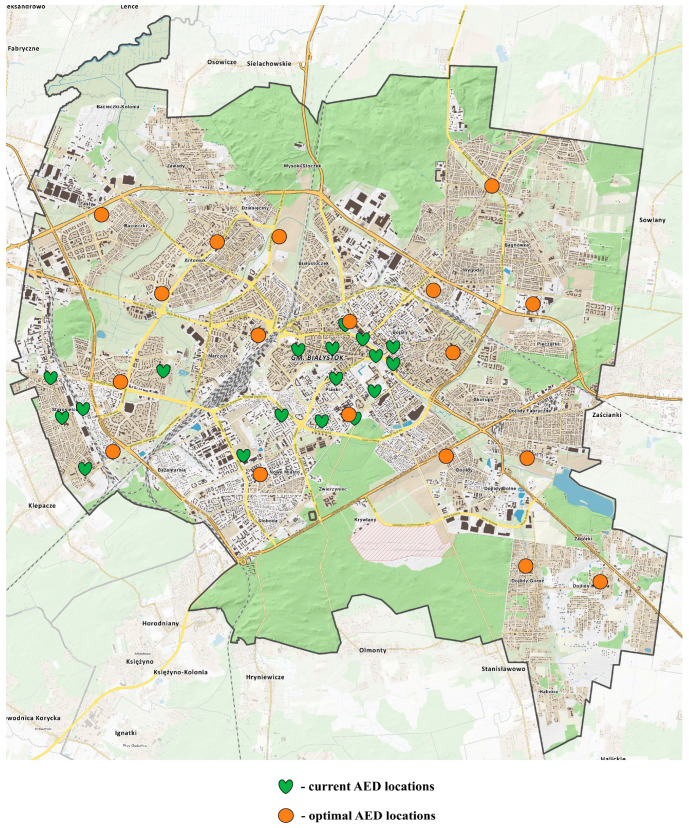
Map of current and proposed AED locations based on 18-cluster analysis of 766 OHCA cases (residential + public).

Table 1 presents the geographic coordinates of the areas with the highest incidence of OHCA. A total of 766 cases occurred in both public locations and private residences. Cases that occurred in detention centers, sobering stations, nursing homes, hospices, and transport ambulances were excluded from the analysis. The current AED locations (marked with green heart symbols) and proposed optimal AED placements (marked with orange circular symbols) were plotted on a map of Bialystok based on the corresponding geographic coordinates (Figure 2).

OHCA Cases in Public Locations (Number of Clusters = 18).

Table 2 above presents the geographic coordinates of public areas with the highest incidence of OHCA. This includes 136 cases that occurred in public locations such as streets, shops, outpatient clinics, construction sites, hotels, parks, allotment gardens, beaches, workplaces, sewage treatment plants, garages, warming shelters, car repair shops, shopping malls, dormitories, schools, train stations, bus stations, government offices, wholesale produce markets, waste shelters, sports stadiums, former military hangars, libraries, wedding venues, universities, abandoned buildings, and small forested areas.

**Figure 3:**
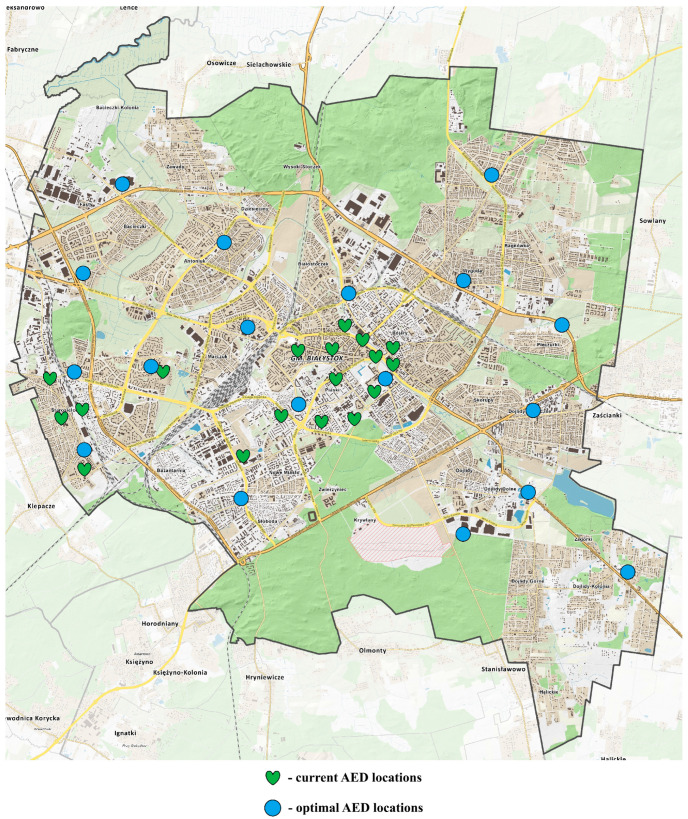
Map of current and proposed AED locations based on 18-cluster analysis of 136 OHCA cases in public locations.

Current AED locations (represented by green heart symbols) and proposed optimal AED placements (represented by blue circular symbols) were mapped onto the layout of Bialystok according to the corresponding geographic coordinates (Figure 3).

A similar analysis was conducted using 36 clusters. In this scenario, 36 optimal points were identified under the assumption of doubling the number of publicly accessible AEDs, either by relocating the existing 18 devices and adding 18 new ones or by maintaining the original 18 locations and designating 36 additional optimal sites.

The analysis revealed that the current distribution of AED devices across Bialystok is suboptimal.

## 4. Discussion

### Use and Distribution of AEDs in Bialystok: A Two-Year Retrospective Analysis

In Bialystok, the use of AEDs by bystanders in cases of OHCA before the arrival of EMS remains infrequent. Over a two-year period, an AED was deployed in only four incidents (0.51% of all cases). Of these, two involved the State Fire Service, one involved detention center personnel, and one involved sobering station staff. Comparable low usage rates were reported in the Silesian Voivodeship, where AEDs were used in just 0.8% of OHCA cases in 2018 [17], and in Poznan, where usage rates were 1.3% between 2018 and 2019 [18]. Slightly higher AED usage by bystanders is observed in the United States (2.2%) and the United Kingdom (5.5%) [19,20].

A statistically significant correlation was observed between ROSC and initial cardiac rhythm. Among patients presenting with VF or pVT, ROSC was achieved in 58.62% of cases. Conversely, for patients with non-shockable rhythms, such as asystole or PEA, ROSC was observed in only 26.56% of cases. These findings are consistent with European data, where ROSC rates were 58% for shockable rhythms and 26% for non-shockable rhythms [21]. Similar outcomes were recorded in Katowice in 2018, where ROSC was noted in 51.3% of patients presenting with an initial rhythm of VF/pVT [17].

Studies have indicated that earlier rhythm analysis following OHCA increases the likelihood of detecting a shockable rhythm. AEDs, which typically assess rhythm an average of 3 min and 43 s earlier than EMS, detected VF/VT in 63.33% of suspected OHCA cases. The remaining 36.67% had non-shockable rhythms [13].

In Europe, OHCAs most commonly occur at home. In 2014 and 2017, 69.4% and 70.2% of OHCAs occurred in residential settings, respectively [21,22]. Our analysis revealed that 80.05% of the OHCA incidents in Bialystok occurred at home. Approximately 9.78% of incidents occurred on public streets. In Upper Silesia, 74.7% of OHCAs in 2018 occurred at home, while 6.3% occurred on streets [23]. This trend was mirrored in the United Kingdom, where, in 2021, 83.2% of OHCAs occurred at home and 10.4% on the street [19].

In Bialystok, EMS was most commonly alerted by family members (67.47%), followed by witnesses (11.94%) and other services, such as the police, fire departments, municipal guards, or other EMS units (4.96%). In the Swietokrzyskie Voivodeship, 76.3% of EMS calls were made by family members in 2016 [24].

The leading reason for EMS dispatch in confirmed OHCA cases was the absence of breathing (41.80%). Unconscious patients who retained spontaneous breathing accounted for 17.92% of calls. Other reasons included dyspnea (10.80%), syncope (6.86%), and chest pain (3.05%). OHCAs represented 1.6% of all EMS dispatches related to life- or health-threatening emergencies in Bialystok. In Poznan in 2015, apnea and unconsciousness accounted for 30.63% and 30.32% of calls, respectively. Other reasons included respiratory conditions (13.17%) and chest pain (5.08%). In Poznan, OHCAs accounted for 1.07% of all EMS dispatches [25].

As of February 1, 2023, Bialystok had 18 publicly accessible (outdoor) AEDs and 28 devices located inside stores, workplaces, private enterprises, and public institutions. We analyzed 766 OHCA cases in Bialystok and mapped each case to a specific geographic coordinate. Cluster analysis was performed based on two groups: all public and residential locations and public locations only. This approach allowed us to identify the optimal AED deployment points to minimize the distance to past OHCA locations. Maps and optimal AED sites were generated for each group, and only publicly accessible AEDs were included in the comparative analysis. Results from all four scenarios revealed that current AED placements are suboptimal based on spatial analysis.

Currently, AED placement in Bialystok is unregulated and largely dependent on public and private initiatives. According to the 2021 ERC guidelines, AEDs should be recommended for sites with at least one OHCA in the past five years, especially in high-traffic areas where an OHCA is likely to be promptly recognized. In residential neighborhoods, past experiences and community characteristics should guide AED placement [3,14,15]. According to the ERC and the American Heart Association (AHA), at least one AED should be available in every public transport facility, including trains, bus stations, and airports [26]. Mathematical modeling of AED deployment may improve accessibility and OHCA survival outcomes in accordance with ERC and AHA recommendations [27].

A study conducted in Paris in 2018 compared two AED deployment strategies. The first was a grid-based approach with devices spaced 200–2000 m apart. The second used landmarks, such as post offices, metro stations, and pharmacies. The landmark-based strategy resulted in shorter average distances between AEDs and OHCA incidents. Public bicycle stations were identified as the most effective locations [28].

In Poland (2008–2018), AEDs were most commonly placed in supermarkets and shopping malls. Location decisions were based on proximity to medical facilities and the density of customers. Among users, 80% were trained individuals such as security personnel, police officers, swimming pool staff, public transport drivers, hotel personnel, and medical staff. Only 20% of AED uses involved lay bystanders, and even then, medical professionals or healthcare students comprised the majority [26].

Our spatial analysis identified “PSS Spolem” grocery stores in Bialystok as potentially optimal AED locations. Of the 36 recommended AED points (including residential OHCA cases), 11 (31%) matched existing PSS Spolem store locations, representing about 46% of all PSS Spolem branches. These stores are well known and frequently visited, facilitating rapid access to AEDs. Staff training in Basic Life Support (BLS) with AED use could further improve outcomes. Similar conclusions were drawn from a Taiwanese study, which recommended AED placement at bus stops or in widely recognized stores [29].

The low bystander AED usage rate may be attributed to several factors, including insufficient AED availability, suboptimal placement relative to OHCA locations, inadequate public education, limited 24-h access, poor signage, and difficulty in locating devices. During the study period, emergency dispatchers lacked real-time information on nearby AEDs because these data were not integrated into the dispatch system. Dispatcher instructions typically focus on CPR alone. Moreover, since most OHCAs occur in residential areas and AEDs are predominantly located in public spaces, accessibility remains limited [22,30,31].

Our findings are broadly consistent with preliminary trends reported by the forthcoming EuReCa THREE registry (in press), which confirms a persistently high proportion of residential OHCA cases across Europe, low public-access AED use, and modest rates of bystander-initiated CPR. However, the observed incidence of OHCA (133.1 per 100,000 annually) is slightly below the reported European average. Discrepancies may result from differences in EMS dispatch coding, data completeness, or regional AED availability [32,33].

A notable limitation of our study is the substantial proportion (37.1%) of OHCA records in which AED usage status was not clearly documented. This lack of confirmation may bias estimates of public-access defibrillation use and underrepresent actual bystander involvement. Similarly, the exclusion of incomplete or ambiguously coded cases may have led to an underestimation of overall OHCA incidence in Bialystok.

Increasing the number of AEDs in a city correlates with higher usage rates and improved survival after OHCA. A French study showed survival rates of 18% in areas with high AED density versus 8% in low-density areas [34]. Decreasing the distance between AEDs and potential OHCA locations significantly enhances the chances of survival. If an AED was located within 200 m of an OHCA site, the 30-day survival rate doubled [35].

AEDs should be universally accessible and placed in locations with 24/7 public access. In retail environments, devices should be externally installed. AEDs must be clearly and intuitively marked. In addition to international signage, labels in Polish (e.g., “defibrylator”) should be included. To improve pre-EMS defibrillation, innovative strategies such as mobile phone applications, social media alerts, SMS notifications, AED delivery via drones, and community responder programs involving trained volunteers should be adopted.

While our use of K-means clustering allowed us to empirically detect spatial OHCA hotspots, this method is limited by its reliance on Euclidean distance and the absence of real-world constraints. It does not consider pedestrian access, road networks, or the time required to retrieve an AED. Therefore, future work should incorporate network-based spatial optimization tools, such as the Location-Allocation method in ArcGIS Pro, which allows modeling based on actual travel paths, response time, and demand weighting.

Dispatch-assisted CPR (DA-CPR) is one of the most effective interventions for improving OHCA outcomes. Through real-time guidance, dispatchers can significantly increase the likelihood of bystander CPR, especially in communities with low training coverage. Despite its proven efficacy, the dispatch system in Bialystok currently provides CPR instructions but not AED detection or use instructions. Improving dispatcher training on AED location and implementing structured CPR algorithms combined with dispatcher-assisted AED use could significantly improve community response and patient survival [36].

This study has several limitations. First, the K-means clustering method used relies on Euclidean distances and does not account for real-world constraints, such as road networks or travel times. Second, the analysis did not incorporate population density, socioeconomic status, or temporal variations in the incidence of OHCA. Third, the dispatch system did not provide real-time AED location integration during the study period, which limited the public’s ability to retrieve defibrillators.

Furthermore, our choice of K-means clustering, while computationally efficient, did not account for walking paths, accessibility, or response times. More advanced methods, including Location-Allocation analysis in ArcGIS Pro, should be applied in future research to derive actionable recommendations for AED placement based on actual movement networks and emergency access time.

Although we did not conduct a systematic sensitivity analysis of different K values, preliminary inspection suggests that the main OHCA hotspots persist even with minor adjustments in cluster number. This observation supports the stability and validity of our findings, although further studies using formal cluster validation methods are warranted.

Importantly, according to EMS records, none of the publicly accessible AEDs in Bialystok were used during the study period. This severely limits our ability to evaluate whether device placement was appropriate.

## 5. Conclusions

Our study reveals substantial spatial and systemic shortcomings in the emergency response to OHCA in Bialystok. AEDs remain significantly underutilized, particularly in residential areas where most arrests occur, and their placement does not correspond to actual OHCA risk zones.

During the study period, the EMS dispatch center in Bialystok operated without real-time integration of AED location data. Dispatchers were unable to inform callers of the nearest AED, and PAD locations were not visible in their system. Instructions focused solely on initiating CPR, which limited the potential for public AED use.

BLS training in the general population remains low in Poland, with substantial geographic disparities. Urban populations benefit from better access to education and AED awareness programs, while rural or peripheral communities remain underserved and underprepared to respond to cardiac emergencies.

Our cluster analysis, based on straight-line distances, identified concentrated OHCA zones but lacked integration with population density, socioeconomic vulnerability, or real travel-time metrics. Future analyses should incorporate these elements for more accurate public health planning.

These findings have important practical implications. Locally, they advocate for a coordinated AED placement policy, integrated dispatch systems with AED geolocation, and widespread implementation of DA-CPR protocols. Globally, this work reinforces the urgent need for data-driven, spatially aware strategies for OHCA preparedness—strategies that combine technology, infrastructure, and public engagement.

## Figures and Tables

**Table 1:** Geographic coordinates of the most frequent OHCA locations (residential + public, *n* = 766) (number of clusters = 18).

	Latitude	Longitude	Number of Cases	Percentage (%)
1	53.14379	23.18232	41	5.35
2	53.14407	23.10618	27	3.52
3	53.12881	23.09460	63	8.22
4	53.12441	23.16043	75	9.79
5	53.11302	23.13475	64	8.36
6	53.15314	23.14322	18	2.35
7	53.11637	23.18424	21	2.74
8	53.15184	23.12268	56	7.31
9	53.13243	23.18411	64	8.36
10	53.13866	23.15582	87	11.36
11	53.09957	23.20651	11	1.44
12	53.11723	23.09204	42	5.48
13	53.09454	23.23064	6	0.78
14	53.16190	23.19955	21	2.74
15	53.13655	23.13333	102	13.32
16	53.15623	23.09176	21	2.74
17	53.14155	23.21188	21	2.74
18	53.11914	23.21009	26	3.39

**Table 2:** Geographic coordinates of public locations with the highest incidence of OHCA (number of clusters = 18).

	Latitude	Longitude	Number of Cases	Percentage (%)
1	53.12904	23.17078	21	15.44
2	53.15216	23.12639	11	8.08
3	53.12356	23.21381	3	2.21
4	53.11095	23.21006	5	3.68
5	53.13670	23.13129	20	14.71
6	53.14301	23.16195	22	16.18
7	53.10884	23.12874	6	4.41
8	53.09786	23.23850	1	0.74
9	53.13016	23.08261	3	2.21
10	53.16336	23.09307	5	3.68
11	53.09998	23.19562	2	1.47
12	53.14455	23.19330	6	4.41
13	53.14717	23.08328	2	1.47
14	53.12519	23.14704	15	11.03
15	53.11686	23.08648	5	3.68
16	53.16364	23.20008	2	1.47
17	53.13684	23.22438	2	1.47
18	53.13160	23.10494	5	3.68

## Data Availability

The data will be made available upon request from the corresponding author.
